# (Dis)trust in doctors and public and private healthcare institutions in the Western Balkans

**DOI:** 10.1111/hex.13562

**Published:** 2022-07-03

**Authors:** Driton Maljichi, Blerim Limani, Troy E. Spier, Violeta Angjelkoska, Sanja Stojković Zlatanović, Drita Maljichi, Iliriana Alloqi Tahirbegolli, Bernard Tahirbegolli, Ahmed Kulanić, Irida Agolli Nasufi, Milica Kovač‐Orlandić

**Affiliations:** ^1^ Social Science Department University St. Cyril and Methodius Skopje North Macedonia; ^2^ Liberal Arts Department American University of Middle East Kuwait City Kuwait; ^3^ English Department Universidad San Francisco de Quito Quito Ecuador; ^4^ Faculty of Communication and IT American University of Europe—FON Skopje North Macedonia; ^5^ Social Science Department Institute of Social Sciences Belgrade Serbia; ^6^ Management in Tourism and Hospitality Department Pjeter Budi College Prishtina Kosovo; ^7^ Department of Health Institutions and Services Management | Nursing Department Heimerer College Prishtina Kosovo; ^8^ Hematology Clinic University Clinical Center of Kosovo Prishtina Kosovo; ^9^ National Sports Medicine Centre Prishtina Kosovo; ^10^ Institute for Bosniak Studies Sarajevo Bosnia and Herzegovina; ^11^ Research Center for Neighbouring Countries and Regions Istanbul Ticaret University Istanbul Turkey; ^12^ Social Science Department University of Tirana Tirana Albania; ^13^ University Donja Podgorica Montenegro

**Keywords:** healthcare institutions, medical doctors, trust, Western Balkans

## Abstract

**Introduction:**

Considering the geopolitical changes in the six Western Balkan countries—Albania, Bosnia and Herzegovina, Kosovo, Montenegro, North Macedonia and Serbia—over the last three decades, particularly as it concerns the progress and changes in the healthcare systems, we argue that there is a need for a detailed analysis of people's trust in those healthcare systems and healthcare providers.

**Methods:**

In this cross‐sectional, intercountry study, we examine the trust trends of Western Balkans citizens in medical doctors and public and private healthcare institutions from 25 July 2021 to 30 October 2021, with 3789 participants using a self‐reported questionnaire, and Google Forms. Snowball sampling is used to collect data from six Western Balkans countries: Albania, Bosnia and Herzegovina, Kosovo, Montenegro, North Macedonia and Serbia.

**Findings:**

The primary findings of our study show that citizens in the Western Balkans have a low level of trust in their healthcare system (*X̄* = 4.3/10). Medical doctors working in private healthcare institutions, on the other hand, are afforded a higher level of trust (*X̄* = 6.6/10) than those working in public healthcare institutions (*X̄* = 5.7/10). In the event that they or their family members need to visit a health institution, half of the study participants would choose private healthcare institutions over public ones. We found a statistically significant difference between countries on the mean points from the questions concerning one's trust in the healthcare system, private healthcare institutions and medical doctors working in public and private sectors (*p* < .05).

**Conclusion:**

Despite its limitations, this study is the first cross‐sectional research on the ‘trust interface’ among western Balkan citizens, revealing that they have low trust in their healthcare systems.

**Public Contribution:**

The information in this manuscript was gathered on the level of 3789 citizens from six Western Balkan countries. Before we began collecting data, we conducted a piloting procedure with 40 citizens who were clients of health institutions to validate the data collection questionnaire.

## 1 INTRODUCTION

Trust is considered an important component of long‐term relationships with medical providers and health insurers, therapeutic adherence to prescribed medications, and it may be related to the extent to which patients seek routine medical care.^1^, ^2^, ^3^ In fact, researchers have found various correlations of relevant factors in trust in healthcare systems, both at the national and cross‐national levels.^4^, ^5^, ^6^ Furthermore, Ozawa and Sripad^7^ have also demonstrated various approaches to measuring trust in institutions and among health workers through the use of different instruments and variables. Others argue that, rather than general assessments of public trust in healthcare, questions about specific aspects of microlevel healthcare, such as professional expertize and the doctor–patient relationship, should be asked.^8^

Generalized trust in healthcare systems and trust specifically in healthcare institutions are manifested as relational processes that are always an input to and the result of a relationship, and that can change longitudinally depending on country governance, healthcare investments and expenditures.^6^, ^9^, ^10^, ^11^ According to Adam and Donelson,^11^ the partnership's strength and ongoing motivation to engage in positive changes to healthcare systems demonstrate trust‐building through recurring reciprocity cycles. However, even in wealthier countries, many of which are believed to have well‐consolidated healthcare systems, people's trust in healthcare varies. For instance, van der Schee et al.^6^ recognized that Germans have lower trust in their medical system compared with citizens of England, Wales and the Netherlands. Zhao et al.^9^ discovered, among the 31 countries surveyed in their study, that Belgium had the highest degree of trust; Poland, the lowest. Moreover, Jovell et al.^12^ found that trust in various public institutions varies, but healthcare institutions rank very highly in the minds of Spanish citizens. Furthermore, patients' willingness to commit to medication, regardless of their economic situation, is influenced by physician trust levels.^5^ Frankel et al.^13^ emphasize the importance of physicians cultivating and maintaining an ongoing discipline of reflecting on interpersonal interactions and the quality of relationships with patients, other members of the medical staff and one another, both individually and collectively.

Considering the geopolitical changes in the six western Balkan countries over the last three decades, particularly the progress and changes in healthcare systems, we argue that there is a need for a detailed analysis of people's trust in healthcare systems and healthcare providers. One might argue that the significance of the present study is also affected by a global pandemic the COVID‐19, which has changed the social fabric as we used to know it, and in turn, additionally changed people's perception and trust in healthcare institutions. The present study aims to investigate citizens' level of trust in public and private healthcare institutions and medical doctors in the Western Balkans.

## 2 METHODS

A self‐reported questionnaire is used to collect information on the level of six western Balkan countries' citizens (3789 participants) aged 18 through 70, from 25 July 2021, through 30 October 2021, and they were recruited through the snowball sampling technique.^14^ An online version of the questionnaire was also made using Google Forms and piloted among 40 participants in Albanian. The international research team reviewed the questionnaire for acceptability in their country/cultural context and discussed all suggested changes until a consensus was reached. After piloting, the internal consistency of the questionnaire is calculated (Cronbach's *α* = .844). Since the questionnaire was originally written in Albanian, an identical version of the questionnaire was translated into Macedonian, Montenegrin, Serbian and Bosnian for usage in their respective countries, using the double‐forward–backward method.^15^ Internal consistency was also relatively high for the versions of the questionnaire provided in Macedonian (Cronbach's *α* = .931), Montenegrin (Cronbach's *α *= .913), Serbian (Cronbach's *α *= .909) and Bosnian (Cronbach's *α *= .926).

We asked respondents for the following demographic information: age, gender, employment status, education, income status, marital status and previous receive of health service. Ethnicity was self‐reported in response to the fixed‐choice questions based on the Census 2011 in all the six western Balkan countries. To assess trust in the healthcare system, healthcare institutions and physicians, we asked respondents to rate their agreement with statements (e.g., ‘How much do you trust…’), and responses were on a 10‐point scale where 1 indicated ‘no trust at all’ and 10 indicated ‘a lot of trust’. Given the study's methodology and the fact that the study sample's health literacy remains unknown, the term *healthcare system* is used to encompass both public and private health institutions to facilitate the understanding of concepts by respondents from various backgrounds in the present study. Two contributions include the fact that most Balkan countries have mandatory national health insurance and most healthcare institutions are publicly owned.^16^

The estimated population of Albania, Bosnia and Herzegovina, Kosovo, Montenegro, North Macedonia and Serbia is 13.1 million, of which approximately 75% are over the age of 18. Therefore, drawing from this population with a 95% confidence level and confidence interval (CI) of 2, a minimum sample size of 2401 persons was determined. Thus, the total number of participants in the present study significantly exceeded the minimum sample size.

The study protocol was approved by the ethical commission of Heimerer College in Pristina, Kosovo and the procedures of this study complied fully with the provisions of the Declaration of Helsinki regarding Research on Human Participants. All subjects provided informed consent electronically before registration, and the informed consent page presented two options: *yes* and *no*. Only those subjects who selected the former could proceed with the questionnaire.

Data analysis was performed using the Statistical Package for the Social Sciences (SPSS version 21.0).^17^ Descriptive analysis was utilized to present general statements about the data. For count data, frequencies and percentages were used. Continuous variables are summarized with mean ± SD or mean and SE. The distribution of normality was evaluated using Kolmogorov–Smirnov tests, while the independent samples *t*‐test, one‐way analysis of variance post‐hoc Tukey test, *χ* ^2^ test and adjusted linear regression (*β* and 95% CI) were used to analyse variables. For all statistical tests, a *p* < .05 was considered statistically significant.

## 3 RESULTS

Sixty‐five percent of the participants were female. Twenty‐seven percent of the participants were between the age of 31 and 40. More than half of the participants in the study sample received a university education, and the majority were married with an economic status equivalent to the middle class. The participants' distribution by country and ethnicity is demonstrated in Table 1.

**Table 1:** Sociodemographic characteristics of the participants (*n* [%])

Country			
Albania		601	15.9%
Bosnia and Herzegovina		700	18.5%
Kosovo		633	16.7%
Montenegro		422	11.1%
North Macedonia		624	16.5%
Serbia		809	21.4%
Gender			
Male		1306	34.5%
Female		2483	65.5%
Age range (in years)			
18–30		936	24.7%
31–40		1027	27.1%
41–50		913	24.1%
51–60		633	16.7%
61–70		281	7.4%
Education			
Secondary school		1152	30.4%
University		1964	51.9%
MSc or PhD		673	17.7%
Ethnicity			
Albanian		1470	38.8%
Serbian		1079	28.5%
Bosnians		389	10.3%
Macedonian		316	8.3%
Montenegrin		198	5.2%
Croatian		62	1.6%
Slovenian		54	1.4%
Hungarian		31	0.8%
Turks		26	0.7%
Roma		13	0.3%
Other		151	4.0%
Marital status			
Single		991	26.2%
Married		2446	64.6%
Divorced		240	6.3%
Widowed		112	3.0%
Income			
Low		623	16.4%
Medium		2588	68.3%
Good		578	15.3%
Living setting			
Urban		3051	80.5%
Rural		738	19.5%

Conversely, the participant's most recent visit to a health facility ranged considerably from 0 to 360 months ago, as indicated in Table 2.

**Table 2:** Most recent visit to a health facility

	Range (in months)		Mean	Std. deviation
	Minimum	Maximum		
Albania	1.00	244.00	19.06	35.79
Bosnia and Herzegovina	0.00	160.00	21.90	39.64
Kosovo	1.00	96.00	10.47	19.61
Montenegro	1.00	160.00	20.83	37.01
North Macedonia	1.00	360.00	38.71	91.39
Serbia	1.00	150.00	24.28	37.45
Total	0.00	360.00	22.70	49.52

**Figure 1:**
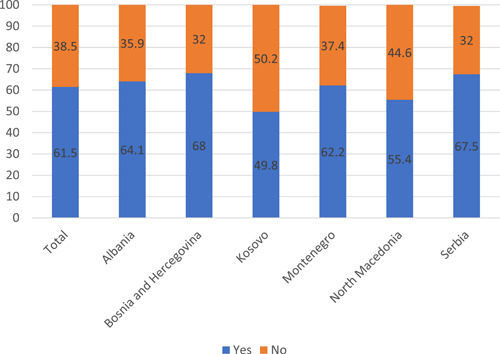
Rates of negative experiences at a healthcare institution (last year).

Sixty‐one percent of the study sample had at least one negative experience during the last 12 months while they or their close relative(s) received a medical service in a healthcare institution. The highest prevalence of negative experiences was found in Bosnia and Herzegovina (68%); the lowest, in Kosovo (49.8%). The participants' affirmative and negative responses to the following question are listed in Figure 1: ‘During the last 12 months, have you or any of your close relatives had any negative experiences while receiving treatment in a healthcare institution?’

**Figure 2:**
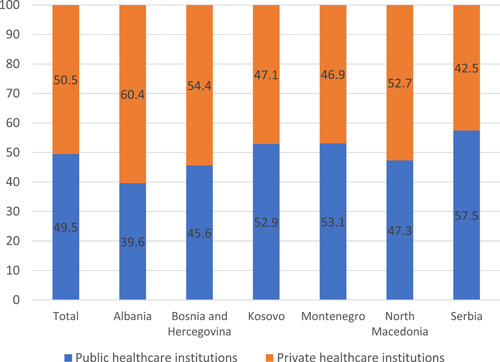
Preference for public or private institutions (%).

There was a statistically significant difference in the participants' preference to visit private healthcare institutions or public healthcare institutions by country (*p* < .05; Table 3). Additionally, the survey data show that half of the participants prefer private healthcare institutions over their public counterparts, in case they or their family members were faced with a need to visit them. More than 60% of Albanian citizens prefer private healthcare institutions, while 57% of Serbian citizens prefer a public one. Both preferences can be seen in Figure 2.

**Table 3:** Comparison by country preferences for public or private healthcare institutions

		Albania, *n* (%)	Bosnia and Herzegovina, *n* (%)	Kosovo, *n* (%)	Montenegro, *n* (%)	North Macedonia, *n* (%)	Serbia, *n* (%)	
Preference to visit firstly	Public healthcare institutions	238 (6.3)	319 (8.4)	335 (8.8)	224 (5.9)	295 (7.8)	465 (12.3)	*χ* ^2^ = 54.849, *p* < .0001
	Private healthcare institutions	363 (9.6)	381 (10.1)	298 (7.9)	198 (5.2)	329 (8.7)	344 (9.1)	

Furthermore, there is a statistically significant difference between countries on the mean points from the questions concerning citizens' trust in the healthcare system, private healthcare institutions and medical doctors working in public and private sectors (*p* < .05), as found in Table 4. The post‐hoc Tukey Test showed a statistically significant difference in the mean points concerning trust in the healthcare system between Kosovo and all other countries in the Western Balkans, between Albania and Serbia, between Montenegro and Bosnia and Herzegovina, between Serbia and North Macedonia and between North Macedonia and Bosnia and Herzegovina (*p* < .05). A statistically significant difference was found also in the mean points concerning citizens' trust in private healthcare institutions between Bosnia and Herzegovina and Kosovo, Albania, Serbia and North Macedonia (*p* < .05). Moreover, there is a statistically significant difference in the mean point concerning trust toward medical doctors working in the public sector between Kosovo and all other countries, between Serbia and North Macedonia and between Bosnia and Herzegovina and North Macedonia. Finally, there is a statistically significant difference between the mean point in trust toward medical doctors working in private sectors, specifically between Kosovo and Albania, Albania and Bosnia and Herzegovina, Serbia and Bosnia and Herzegovina, and North Macedonia and Bosnia Herzegovina (*p* < .05).

**Table 4:** Participants' trust levels in/towards entities and institutions^a^

Trust in/toward…	The healthcare system		Private healthcare institutions		Doctors in public institutions		Doctors in private institutions	
	Mean	Std. error	Mean	Std. error	Mean	Std. error	Mean	Std. error
Albania	4.27	0.11	6.26	0.10	5.59	0.11	6.27	0.10
Bosnia and Herzegovina	3.62	0.10	7.11	0.09	5.38	0.10	6.98	0.09
Kosovo	5.73	0.11	6.66	0.10	6.62	0.11	6.75	0.10
Montenegro	4.20	0.14	6.68	0.13	5.58	0.14	6.68	0.13
North Macedonia	4.32	0.11	6.42	0.10	5.84	0.10	6.51	0.10
Serbia	3.80	0.10	6.56	0.09	5.29	0.10	6.49	0.09
Total	4.29	0.46	6.62	0.04	5.70	0.04	6.61	0.04
	*F* = 49.45, *p* < .0001		*F* = 8.82, *p* < .0001		*F* = 20.61, *p* < .0001		*F* = 6.52, *p* < .0001	

^a^ 1 Indicates ‘no trust at all’ and 10 indicates ‘a lot of trust’.

The participants who prefer public healthcare institutions over their private counterparts, in case they or their family members were presented with the need to visit them, have had higher points on the questions concerning citizens' trust in the healthcare system (mean [SE]: 5.16 [0.07] and 3.44 [0.05], respectively) (*t* = 19.845, *p* < .0001), and trust toward medical doctors working in the public sector (6.560 [0.06] and 4.83 [0.06], respectively) (*t* = 20.507, *p* < .0001). The participants who prefer private healthcare institutions over their public counterparts, in case they or their family members were presented with the need to visit them, had higher points the questions concerning citizens' trust in private healthcare institutions (7.27 [0.05] and 5.96 [0.06], respectively) (*t* = 16.509, *p* < .0001), and trust toward medical doctors working in the private healthcare institutions (7.01 [0.05] and 6.14 [0.06], respectively) (*t* = 11.760, *p* < .0001).

The participants who prefer public healthcare institutions over their private counterparts, in case they or their family members were faced with a need to visit them have had a higher points the questions concerning citizens' trust in the healthcare system, and trust toward medical doctors working in the public healthcare institutions in Albania, Kosovo, Montenegro, Serbia, Bosnia and Herzegovina and Macedonia (*p* < .05). On the other hand, participants who prefer private healthcare institutions over their public counterparts, in case they or their family members were presented with the need to visit them, had higher points on the questions concerning citizens' trust in the private healthcare institutions, and trust toward medical doctors working in the private healthcare institutions in Albania, Kosovo, Montenegro, Serbia, Bosnia and Herzegovina and Macedonia (*p* < .05).

There was no statistically significant difference based on the participants' gender regarding points of the questions concerning citizens' trust in the healthcare system and trust toward medical doctors working in the public healthcare institutions (*p* > .05). Female participants were found to have higher points compared to male counterparts on the question regarding trust in private healthcare institutions (mean [SE]: 6.74 [0.05] and 6.39 [0.07], respectively) (*t* = 4.141, *p* < .0001), and trust toward medical doctors working in the private healthcare institutions (6.69 [0.05] and 6.47 [0.07], respectively) (*t* = 2.608, *p* = .009).

When analysed separately by country, females had higher points compared to males on the question regarding trust in private healthcare institutions in Albania, and trust toward medical doctors working in the public healthcare institutions in Bosnia and Herzegovina (*p* < .05; Table 5).

**Table 5:** Participants' trust levels in/towards the health system based on some sociodemographic characteristics

	Total		Albania		Bosnia and Herzegovina		Kosovo		Montenegro		North Macedonia		Serbia	
	Mean ± SD	*p*‐value	Mean ± SD	*p*‐value	Mean ± SD	*p*‐value	Mean ± SD	*p*‐value	Mean ± SD	*p*‐value	Mean ± SD	*p*‐value	Mean ± SD	*p*‐value
Gender															
M		4.30 ± 2.83	.971	4.28 ± 2.83	.944	3.38 ± 2.52	.096	5.67 ± 2.71	.666	4.37 ± 3.04	.432	4.54 ± 2.63	.083	3.64 ± 2.80	.242
F		4.29 ± 2.79		4.27 ± 2.58		3.72 ± 2.55		5.77 ± 2.84		4.12 ± 2.89		4.17 ± 2.68		3.88 ± 2.77	
Preference to visit firstly															
Public healthcare institutions		5.16 ± 2.95	<.0001	5.13 ± 2.81	<.0001	4.45 ± 2.85	<.0001	6.75 ± 2.59	<.0001	5.30 ± 3.03	<.0001	5.12 ± 2.75	<.0001	4.49 ± 2.99	<.0001
Private healthcare institutions		3.44 ± 2.36		3.71 ± 2.39		2.93 ± 2.01		4.59 ± 2.56		2.95 ± 2.25		3.60 ± 2.37		2.86 ± 2.14	
Education															
Secondary School		3.82 ± 2.84^a^	<.0001	4.30 ± 2.62	.704	3.54 ± 2.60	.333	5.90 ± 3.01	.802	3.86 ± 2.86	.262	3.66 ± 2.90	.006	3.45 ± 2.80	<.0001
University		4.44 ± 2.78		4.22 ± 2.67		3.78 ± 2.54		5.69 ± 2.83		4.39 ± 2.94		4.48 ± 2.63		3.92 ± 2.68	
MSc or PhD		4.68 ± 2.71		4.46 ± 2.67		3.37 ± 2.33		5.73 ± 2.79		4.20 ± 3.03		4.53 ± 2.45		4.72 ± 2.81^b^	
Income															
Low		3.26 ± 2.59^c^	<.0001	3.20 ± 2.41^c^	<.0001	3.06 ± 2.43^c^	.024	4.38 ± 2.97^c^	.001	3.28 ± 2.79^c^	.010	3.11 ± 2.41^c^	<.0001	3.19 ± 2.64^d^	.003
Medium		4.43 ± 2.76^e^		4.43 ± 2.65		3.68 ± 2.48		5.79 ± 2.73		4.38 ± 2.88		4.38 ± 2.60^e^		4.00 ± 2.77	
High		4.81 ± 2.96		4.75 ± 2.75		3.94 ± 2.85		6.02 ± 2.81		4.53 ± 3.21		5.18 ± 2.75		3.91 ± 2.91	

Abbreviations: F, female; M, male.

^a^ In the post hoc Tukey analyses, those with secondary school education had lower points compared to those with university or postgraduate education.

^b^ In the post hoc Tukey analyses, those with MSc or PhD education had a higher points compared to those with secondary school or university education.

^c^ In the post hoc Tukey analyses, those with low income had a lower points compared to those with medium and high‐income status.

^d^ In the post hoc Tukey analyses, those with low income had a lower points compared to those with medium income status.

^e^ In the post hoc Tukey analyses, those with medium income had a lower points compared to those with high‐income status.

There was no statistically significant difference in gender concerning participants' preference to visit private healthcare institutions or public healthcare institutions (*χ* ^2^ = 1.941, *p* = .164). Furthermore, no statistically significant differences were found in the analysis conducted individually for each country's participants' preference to visit private or public healthcare institutions by gender (*p* > .05).

The higher level of trust in medical doctors seemed to be associated with the doctor's age, with those between the ages of 46 and 55 having the highest level of trust (46.4%), followed by those aged between 36 and 45 having the second‐highest level of trust (22.3%). On the other hand, distrust in medical doctors was associated with the doctor's age, with those aged 23–35 (66.3%) having the highest distrust, followed by those already of retirement age (13%).

The following predictor factors showed a statistically significant impact in the adjusted linear regression analysis for the dependent variable trust in the healthcare system: the participants' age (*β*: −.073, 95% CI: −0.122 to −0.023, *p* = .004), the participants' educational level (*β*: .135, 95% CI: 0.056–0.215, *p* = .001), the participants' economic status (*β*: .196, 95% CI: 0.086–0.306, *p* < .0001), the level of trust in private healthcare institutions (*β*: .062, 95% CI: 0.037–0.086, *p* < .0001) and the level of trust in medical doctors working in public institutions (*β*: .641, 95% CI: 0.617–0.665, *p* < .0001; Table 6). Table 6 also shows the regression analysis results for the dependent variable—trust in the healthcare system—for each Balkan country separately.

**Table 6:** Adjusted linear regression analysis by country^a^

	Total		Albania		Bosnia and Herzegovina		Kosovo		Montenegro		North Macedonia		Serbia	
	*β* (95% CI)	*p*‐value	*β* (95% CI)	*p*‐value	*β* (95% CI)	*p*‐value	*β* (95% CI)	*p*‐value	*β* (95% CI)	*p*‐value	*β* (95% CI)	*p*‐value	*β* (95% CI)	*p*‐value
Age group	−0.068 (−0.380 to 0.462)	.007	–		–		–		–		–		–	
Male gender	–		–		–		–		–		−0.376 (−0.669 to −0.082)	.012	–	
Education level	0.189 (0.109–0.408)	<.0001	–		–		–		0.262 (0.029–0.496)	.028	–		0.328 (0.160–0.497)	<.0001
Income	0.297 (0.186–0.408)	<.0001	–		0.314 (0.062−0.567)	.015	0.372 (0.101–0.643)	.007	–		0.461 (0.205–0.717)	<.0001	–	
Preference of public healthcare institutions over their private counterparts	−0.805 (−0.943 to −0.668)	<.0001	−0.958 (−1.270 to −0.646)	<.0001	−0.603 (−0.932 to −0.275)	<.0001	−1.063 (−1.396 to −0.730)	<.0001	−1.002 (−1.378 to −0.627)	<.0001	−0.691 (−1.012 to −0.370)	<.0001	−0.665 (−0.980 to −0.350)	<.0001
Level of trust in private healthcare institutions	0.117 (0.091–0.143)	<.0001	0.187 (0.127–0.246)	<.0001	0.111 (0.046–0.177)	.001	0.169 (0.106–0.233)	<.0001	0.104 (0.031–0.176)	.005	0.126 (0.063–0.189)	<.0001	0.105 (0.048–0.162)	<.0001
Level of trust in medical doctors working in public healthcare institutions	0.653 (0.628–0.677)	<.0001	0.640 (0.583–0.697)	<.0001	0.539 (0.479–0.599)	<.0001	0.646 (0.584–0.708)	<.0001	0.725 (0.655–0.794)	<.0001	0.640 (0.578–0.702)	<.0001	0.643 (0.592–0.694)	<.0001
Constant	0.041 (−0.380 to 0.462)	.847	1.063 (0.421–1.705)	.001	0.235 (−0.578 to 1.034)	.564	1.096 (0.206–1.986)	.016	0.177 (−0.803 to 1.157)	.723	0.499 (−0.360 to 1.359)	.254	−0.232 (−0.909 to 0.445)	.501

Abbreviation: CI, confidence interval.

^a^ Only variables that had a significant effect in the model are presented here.

## 4 DISCUSSION

The primary findings of our study show that citizens in the Western Balkans have a low level of trust in their respective healthcare systems (*X̄ *= 4.3/10). Medical doctors working in private healthcare institutions, on the other hand, have a higher level of trust (*X̄* = 6.6/10) than those working in public healthcare institutions (*X̄* = 5.7/10). In the event that they or their family members need to visit a hospital, half of the study participants indicated that they would choose private healthcare institutions over their public counterparts. Furthermore, the majority of them experienced at least one negative experience when receiving a service or treatment in a health facility for themselves or close family during the previous 12 months.

In the present study, we have discovered specifically that, among the citizens of the Western Balkans, those residing in Serbia have the lowest trust in the healthcare system overall (3.8/10) and in medical doctors working in public healthcare institutions (5.3/10), while citizens in Albania have the lowest trust in private healthcare institutions (6.3/10) and in medical doctors working in the private sector (6.3/10).

In contrast to this, a study undertaken by Lazarevik and Kasapinov in 2015 indicated that over 75% of Macedonian citizens and over 72% of Serbian citizens had high confidence in their doctors.^18^ In their analysis, Blendon et al.^4^ discovered that Switzerland has the highest level of trust in doctors, followed by Denmark and the Netherlands. Only roughly one‐third of Americans indicated they had significant trust in the medical profession's leadership. Groenewegen et al.^19^ discovered in their study that the Dutch citizens have the highest trust in general physicians' good intentions and medical specialists' expertize. However, this trust has shifted slightly over time.

In this way, Lewandowski et al.'s^20^ study suggests that patient trust in a physician and social trust in payers and hospitals may be related in opposite directions. On the other hand, Gray^21^ lists several consequences that can result from a decline in trust, such as patients seeking a second opinion more frequently, patients will seek services from ‘alternative’ practitioners, and they will want to know ‘the best physicians’ and ‘the best hospitals’. To understand what predicts health provider trust, researchers examined various predisposing sociodemographic factors, such as age, gender, education, income, illness, and so forth.^2^, ^22^, ^23^, ^24^

Additionally, the regression analyses have shown that the following predictive factors are related to lower trust in the healthcare system: age, education level, economic status, level of trust in private healthcare institutions and level of trust in medical doctors working in public institutions.

Finally, Zhao et al.^9^ remark that higher respondent education, urban housing and a lower gross national income all predicted lower healthcare trust. Armstrong et al.^23^ describe similar results in their study, which found that distrust of the healthcare system among Americans was related to age, gender, race, educational levels, family income, access to healthcare and trust in physicians. Furthermore, in any system, providers' behaviours and practices may demonstrate varying levels of trust toward different groups of patients.^25^

## 5 LIMITATIONS

Nonetheless, our findings are limited by our search strategies, which include the use of an online questionnaire as the tool for data‐gathering and the lack of a qualitative analytic design approach. For instance, some researchers have conducted qualitative studies using semi‐structured interviews and focus groups, as well as ethnographic techniques, to assess trust in the health sector.^26^ One of the study's limitations is also the absence of data regarding the Western Balkan countries' health insurance status and health information systems. Because the capacity to provide fast, effective and individualized care is dependent upon how health information systems can or do acknowledge the range of patients, to deliver better insight regarding complex care planning.^27^ Previous research has revealed that having private health insurance is also a significant factor in public trust.^8^ While other well‐designed research could add to this topic by looking into the cause‐and‐effect relationships between factors that reduce and raise trust in health institutions in Western Balkan countries.

## 6 CONCLUSIONS

Despite its limitations, this study, to the best of our knowledge, is the first cross‐sectional research of the ‘trust interface’ among citizens of the nations in the Western Balkans, revealing that they have low trust in their healthcare systems. Although there has not yet been a follow‐up on this study, the COVID‐19 pandemic in which it was conducted reveals important findings concerning the general lack of trust in medical providers in the Western Balkans. Accurate measurements of trust could be used as indicators of healthcare system performance, indicating the need for macrolevel reform.^1^

## AUTHOR CONTRIBUTIONS

*Concept and design*: Driton Maljichi, Blerim Limani, Sanja Stojković Zlatanović, Drita Maljichi, Iliriana Alloqi Tahirbegolli, Bernard Tahirbegolli, Ahmed Kulanić, Irida Agolli Nasufi. *Acquisition, analysis or interpretation of data*: Driton Maljichi, Violeta Angjelkoska, Sanja Stojković Zlatanović, Drita Maljichi, Iliriana Alloqi Tahirbegolli, Bernard Tahirbegolli, Ahmed Kulanić, Irida Agolli Nasufi, Milica Kovač‐Orlandić. *Drafting of the manuscript*: Blerim Limani, Troy E. Spier, Violeta Angjelkoska, Sanja Stojković Zlatanović, Drita Maljichi, Iliriana Alloqi Tahirbegolli, Bernard Tahirbegolli, Ahmed Kulanić, Milica Kovač‐Orlandić. *Critical revision of the manuscript for important intellectual content*: Driton Maljichi, Blerim Limani, Troy E. Spier, Bernard Tahirbegolli, Milica Kovač‐Orlandić. *Statistical analysis*: Irida Agolli Nasufi, Violeta Angjelkoska and Bernard Tahirbegolli. *Administrative, technical or material support*: Driton Maljichi, Troy E. Spier, Violeta Angjelkoska, Sanja Stojković Zlatanović, Drita Maljichi, Iliriana Alloqi Tahirbegolli, Ahmed Kulanić, Irida Agolli Nasufi, Milica Kovač‐Orlandić. *Supervision*: Driton Maljichi, Blerim Limani, and Bernard Tahirbegolli.

## CONFLICT OF INTEREST

The authors declare no conflict of interest.

## ETHICS STATEMENT

The study protocol was approved by the ethical commission of Heimerer College in Pristina, Kosovo, and the procedures of this study complied fully with the provisions of the Declaration of Helsinki regarding Research on Human Participants.

## Data Availability

The data that support the findings of this study are available from the corresponding author upon reasonable request.
